# Impact of right ventricular incision extent on early outcomes after tetralogy of Fallot repair: a two-center retrospective cohort study

**DOI:** 10.3389/fcvm.2025.1702538

**Published:** 2026-01-23

**Authors:** Yun Teng, Jiaxuan Yang, Miao Tian, Shuhua Luo, Jinlin Wu, Ziqin Zhou, Xiaowei Cai, Junfei Zhao, Jimei Chen

**Affiliations:** 1 School of Medicine, South China University of Technology, Guangzhou, Guangdong, China; 2 Department of Cardiac Surgery, Guangdong Cardiovascular Institute, Guangdong Provincial People's Hospital (Guangdong Academy of Medical Sciences), Southern Medical University, Guangzhou, Guangdong, China; 3 The Children’s Heart Center, Department of Cardiac Surgery, West China Second University Hospital, Sichuan University, Chengdu, Sichuan, China; 4 Department of Thoracic and Cardiovascular Surgery, Zhongnan Hospital of Wuhan University, Wuhan, Hubei, China

**Keywords:** pediatric cardiac surgery, perioperative morbidity, postoperative outcomes, right ventricular incision, tetralogy of Fallot (TOF)

## Abstract

**Background:**

The role of right ventricular (RV) incision during tetralogy of Fallot (TOF) repair remains controversial. Although RV incisions facilitate the closure of ventricular septal defects (VSDs) and relieve right ventricular outflow tract (RVOT) obstruction, concerns remain regarding late ventricular dysfunction. Alternative approaches that limit or avoid RV incision have been advocated; however, most evidence derives from single-center retrospective reports, leaving the clinical impact uncertain.

**Method:**

We retrospectively analyzed 237 patients who underwent repair of TOF at two tertiary centers between 2015 and 2019. Patients were stratified into three groups: Group 1 (no RV incision), Group 2 (incision confined to the infundibulum), and Group 3 (incision extending beyond the infundibulum). The primary endpoint was major adverse events (MAEs, defined as in-hospital mortality, need for extracorporeal membrane oxygenation, malignant arrhythmias, delayed sternal closure, reoperation requiring cardiopulmonary bypass, and reintubation). Secondary endpoints included length of intensive care unit (ICU) stay, total hospital stay, ventilation duration, 24-h drainage output, and other postoperative complications. Both crude and propensity score-matched (PSM) analyses were performed.

**Results:**

In crude analyses, delayed sternal closure was more frequent in Group 2 but did not reach statistical significance (*P* = 0.052), while rates of infection and transfusion were higher in Group 3 compared with Group 1. After PSM, differences between Groups 2 and 3 persisted, whereas Group 1 continued to demonstrate more favorable outcomes, likely reflecting more favorable baseline anatomy. Hemodynamic parameters and residual RVOT gradients were comparable across groups after matching.

**Conclusion:**

The extent of RV incision during repair of TOF was associated with distinct perioperative risk profiles; however, rates of major adverse events did not differ significantly after adjustment for baseline imbalances. The more favorable outcomes observed in patients without an RV incision primarily reflected anatomical advantages rather than an intrinsic superiority of the surgical approach. These findings suggest that RV incision should be minimized when technically feasible while ensuring adequate relief of RVOT to ensure procedural safety. Prospective multicenter studies with long-term, imaging-based follow-up are required to determine the impact of incision strategy on RV function, pulmonary regurgitation, and late outcomes.

## Introduction

Tetralogy of Fallot (TOF) is the most common cyanotic congenital heart disease, accounting for 7%–10% of all congenital cardiac malformations (1, 2). Globally, approximately 38,000 newborns are diagnosed with TOF each year. Without surgical intervention, the prognosis is poor, with the 1-year mortality rate approaching 25% and survival beyond the first decade of less than 30% (3). In contrast, contemporary surgical repair has dramatically improved outcomes, with most centers reporting operative mortality rates below 3% (4–8). However, despite improved survival, postoperative morbidity and functional outcomes warrant greater attention.

Standard repair involves closing the malaligned ventricular septal defect (VSD) and relieving the right ventricular outflow tract (RVOT) obstruction. Several operative approaches are available, including preservation or enlargement of the pulmonary valve annulus (PVA), a simple right ventricular (RV) incision, combined right atrial and ventricular incisions, or combined atrial and pulmonary artery (PA) incisions. According to data from both the STS (52%–66%) and EACTS (57.5%) databases, VSD closure via an RV incision with RVOT enlargement remains the most commonly employed global strategy (9, 10). Nevertheless, traditional RV incisions have been associated with late adverse sequelae such as RV dilatation, dysfunction, and tricuspid regurgitation (9, 11–16). In 1960, Hudspeth and colleagues introduced a transatrial and transpulmonary approach that avoids an RV incision, potentially reducing injury to the RV and pulmonary valve and thereby improving early and late outcomes (12, 17–27).

Although several approaches to TOF repair have been described, current evidence regarding the extent of RV incision remains inconsistent. Most available data are derived from retrospective single-center series employing heterogeneous techniques, which limits their generalizability and leaves uncertainty as to whether avoiding or minimizing an RV incision confers a measurable benefit. High-quality multicenter evidence is therefore needed to better inform surgical decision-making.

Accordingly, this multicenter retrospective cohort study was conducted to evaluate the impact of various RV incision strategies on early clinical outcomes after repair of TOF and to identify perioperative risk factors associated with adverse events.

## Methods

### Ethical statement

This study was approved by the Ethics Committees of Guangdong Provincial People's Hospital and West China Second University Hospital (approval number: KY-Q-2021-176). Due to its retrospective design, the requirement for individual informed consent was waived.

### Study design and patient population

We conducted a retrospective, two-center cohort study including patients with tetralogy of Fallot (TOF) who underwent corrective surgery between October 2015 and July 2019. Clinical data were retrieved from the electronic medical record systems of both institutions.

Patients were excluded if they had any of the following: (i) absence of the pulmonary artery; (ii) complex cardiac anomalies (including atrioventricular septal defect, right atrial isomerism, or multiple VSDs); (iii) major extracardiac malformations (such as diaphragmatic hernia, absent sternum, or omphalocele); (iv) unbalanced ventricles precluding biventricular repair; (v) significant genetic abnormalities or syndromes; (vi) infective endocarditis prior to surgery; (vii) stroke within 30 days before surgery; (viii) previous cardiac surgery; or (ix) HIV or hepatitis B infection.

### Surgical technique and grouping

All patients underwent repair via median sternotomy under moderate hypothermic cardiopulmonary bypass (CPB). Myocardial protection was achieved with cold blood cardioplegia. The surgical strategy involved VSD closure, resection of hypertrophic infundibular muscle, pulmonary valve commissurotomy or valvotomy as indicated, and right ventricular outflow tract (RVOT) enlargement using a pericardial or synthetic patch when required. When the pulmonary valve annulus (PVA) *Z*-score was <−3, a transannular patch was used; for *Z*-scores between −3 and −2, the decision was individualized based on intraoperative valve morphology.

Patients were stratified into three groups according to the extent of the right ventricular (RV) incision:

Group 1: no RV incision;
Group 2: incision limited to the infundibulum; and
Group 3: incision extending beyond the infundibulum.

### Statistical analysis

Continuous variables were assessed for normality using the Shapiro–Wilk test. Normally distributed data are presented as mean ± standard deviation (SD) and were compared across groups using one-way analysis of variance (ANOVA), followed by Bonferroni-adjusted pairwise comparisons. Non-normally distributed variables are expressed as median with interquartile range (IQR) and were analyzed using the Kruskal–Wallis test, with *post-hoc* pairwise comparisons performed using Dunn's test with Bonferroni correction.

Propensity score matching (PSM) was performed using covariates including age, weight, oxygen saturation, Nakata index, McGoon ratio, and other relevant baseline characteristics. Matching was conducted using a 1:1 nearest-neighbor algorithm and a caliper width of 0.2 SDs of the logit of the propensity score. Balance between matched groups was assessed using standardized mean difference (SMD), with SMD <0.1 indicating adequate balance.

**Figure 1:**
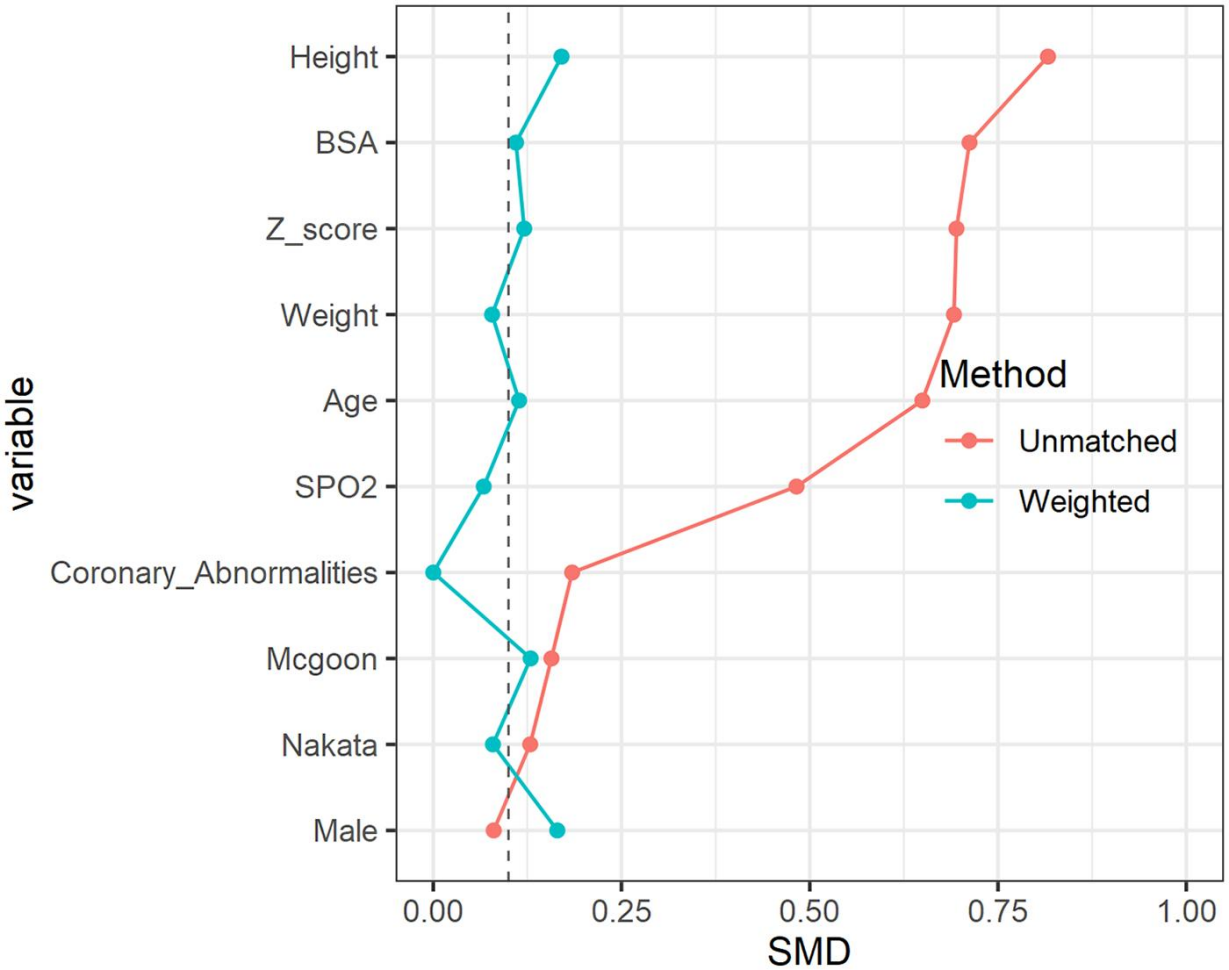
Standardized mean differences (SMDs) before and after propensity score matching. The figure demonstrates covariate balance across groups, with SMD < 0.1 indicating adequate balance. Red dots represent the SMD before matching, while blue dots represent the SMD after matching. The dotted line at SMD = 0.1 indicates the threshold for adequate covariate balance.

Both crude and propensity score-matched results are reported. Supplementary Tables S1–S3 summarize matched baseline characteristics and outcomes, and Figure 1 depicts SMD values before and after matching, demonstrating adequate covariate balance. All analyses were performed using R software (version 3.5.1). A two-sided *p* < 0.05 was considered statistically significant.

## Results

### Patient characteristics

Baseline characteristics are summarized in Table 1. Prior to matching, patients in Group 1 were older, had greater body weight, higher preoperative oxygen saturation, larger pulmonary valve annulus *Z*-scores, and more favorable pulmonary artery indices compared with Groups 2 and 3. After propensity score matching, covariate distributions were well balanced across groups (all SMDs <0.1; Figure 1; Supplementary Table S1).

**Table 1:** Baseline characteristics.

Variables	Overall	No incision	Within infundibulum	Beyond infundibulum	*p*-Value
*n*	237	69	76	92	/
Male (%)	146 (61.6)	44 (63.8)	44 (57.9)	58 (63.0)	0.712
Age [median (IQR)]	11.0 [7.0, 30.0]	31.2 [11.9, 158.5]	13.2 [7.0, 28.8]	8.0 [6.0, 10.3]	<0.001
Height [median (IQR)]	72.0 [67.0, 84.0]	85.0 [74.0, 134.0]	72.5 [68.0, 83.2]	67.5 [65.0, 72.2]	<0.001
Weight [median (IQR)]	8.5 [7.5, 11.0]	11.0 [8.8, 30.0]	8.8 [7.5, 10.5]	8.0 [7.0, 8.5]	<0.001
BSA [median (IQR)]	0.4 [0.4, 0.5]	0.5 [0.4, 1.1]	0.4 [0.4, 0.5]	0.4 [0.4, 0.4]	<0.001
SPO_2_ [median (IQR)]	90.0 [81.0, 95.0]	93.0 [89.0, 97.0]	91.5 [81.8, 95.0]	85.0 [78.0, 94.0]	<0.001
Coronary_abnormalities (%)	4.0 (1.7)	0 (0.0)	1.0 (1.3)	3 (3.3)	0.39
*Z*_score [median (IQR)]	−3.0 [−4.7, −1.5]	−1.5 [−3.2, 0.2]	−3.0 [−5.5, −1.8]	−3.8 [−5.4, −2.7]	<0.001
Nakata [median (IQR)]	206.3 [147.9, 268.6]	194.6 [155.3, 257.0]	194.2 [136.7, 247.1]	223.6 [156.4, 300.1]	0.222
McGoon [median (IQR)]	2.0 [1.6, 2.4]	1.9 [1.7, 2.2]	1.9 [1.5, 2.4]	2.1 [1.6, 2.6]	0.284

### Operative findings

Operative details are presented in Table 2 and Supplementary Table S2. Cardiopulmonary bypass (CPB) and aortic cross-clamp times differed significantly among groups (both *P*'s < 0.001, ANOVA). *Post-hoc* Bonferroni tests showed that CPB times were significantly longer in Group 2 than in Groups 1 and 3, while Group 1 had significantly shorter times. Transannular patching was performed more frequently in Groups 2 and 3 than in Group 1 (*P* < 0.001, chi-square test). After matching, most operative parameters were comparable between groups; however, TAP repair remained significantly more frequent in Groups 2 and 3 compared with Group 1.

**Table 2:** Operative data.

Variables	Overall	No Incision	Within infundibulum	Beyond infundibulum	*p-*Value
TAP (%)	110 (46.4)	0 (0.0)	47 (61.8)	63 (68.5)	<0.001
Extended incision (%)					<0.001
No cut	69 (29.1)	69 (100.0)	0 (0.0)	0 (0.0)	
Within infundibulum	76 (32.1)	0 (0.0)	76 (100.0)	0 (0.0)	
Beyond infundibulum	92 (38.8)	0 (0.0)	0 (0.0)	92 (100.0)	
VSD repair approach (%)					<0.001
RA	155 (65.4)	68 (98.6)	58 (76.3)	29 (31.5)	
RV	61 (25.7)	0 (0.0)	11 (14.5)	50 (54.3)	
RA + RV	20 (8.4)	0 (0.0)	7 (9.2)	13 (14.1)	
PA	1 (0.4)	1 (1.4)	0 (0.0)	0 (0.0)	
CPB time [median (IQR)]	113.0 [98.0, 136.0]	103.0 [93.0, 122.0]	119.5 [109.0, 141.5]	113.0 [96.8, 138.2]	<0.001
ACC time [median (IQR)]	79.0 [60.0, 95.0]	72.0 [60.0, 90.0]	84.0 [75.0, 95.2]	71.5 [57.0, 94.2]	0.001
RV pressure [median (IQR)]	40.0 [34.0, 48.0]	39.0 [33.0, 45.0]	41.0 [35.0, 50.0]	39.0 [34.0, 46.0]	0.232
MPA pressure [median (IQR)]	24.0 [19.0, 31.0]	22.0 [19.0, 28.0]	23.0 [18.0, 30.2]	27.0 [21.0, 34.0]	0.006
System pressure [mean (SD)]	80.8 (14.3)	81.0 (13.8)	78.0 (14.1)	83.0 (14.7)	0.081
Pressure ratio of RV to LV [median (IQR)]	0.5 [0.4, 0.6]	0.5 [0.4, 0.6]	0.5 [0.4, 0.7]	0.5 [0.4, 0.6]	0.126
PG of RVOT [median (IQR)]	13.0 [7.0, 22.0]	14.0 [7.0, 25.0]	15.0 [9.8, 25.2]	10.0 [6.0, 17.0]	0.002

CPB, cardiopulmonary bypass; ACC, aortic cross-clamp; TAP, transannular patch; RV, right ventricle; RA, right atrium; PA, pulmonary artery; MPA, main pulmonary artery; PG, pressure gradient; RVOT, right ventricular outflow tract. Pressures are reported in mmHg; times is in minutes.

### Major adverse events

**Figure 2:**
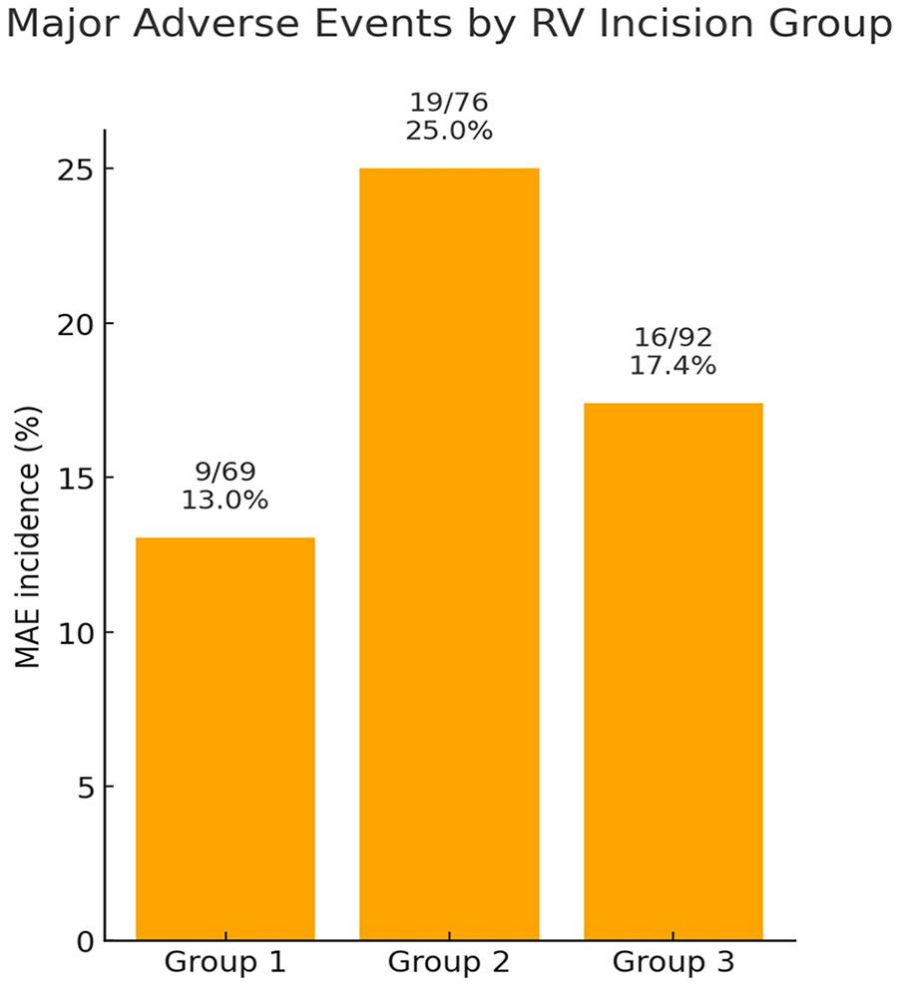
Bar chart illustrating the proportion of patients who experienced MAE in Group 1 (no RV incision), Group 2 (incision confined to the infundibulum), and Group 3 (incision extending beyond the infundibulum). MAE was defined as a composite outcome including in-hospital mortality, extracorporeal membrane oxygenation, malignant arrhythmias, delayed sternal closure, reoperation requiring cardiopulmonary bypass, and reintubation. Values are expressed as percentages with absolute numbers (*n*/*N*) shown above each bar. Although the overall comparison did not reach statistical significance (*P* = 0.184), Group 2 demonstrated the highest incidence of MAE relative to Groups 1 and 3.

**Figure 3:**
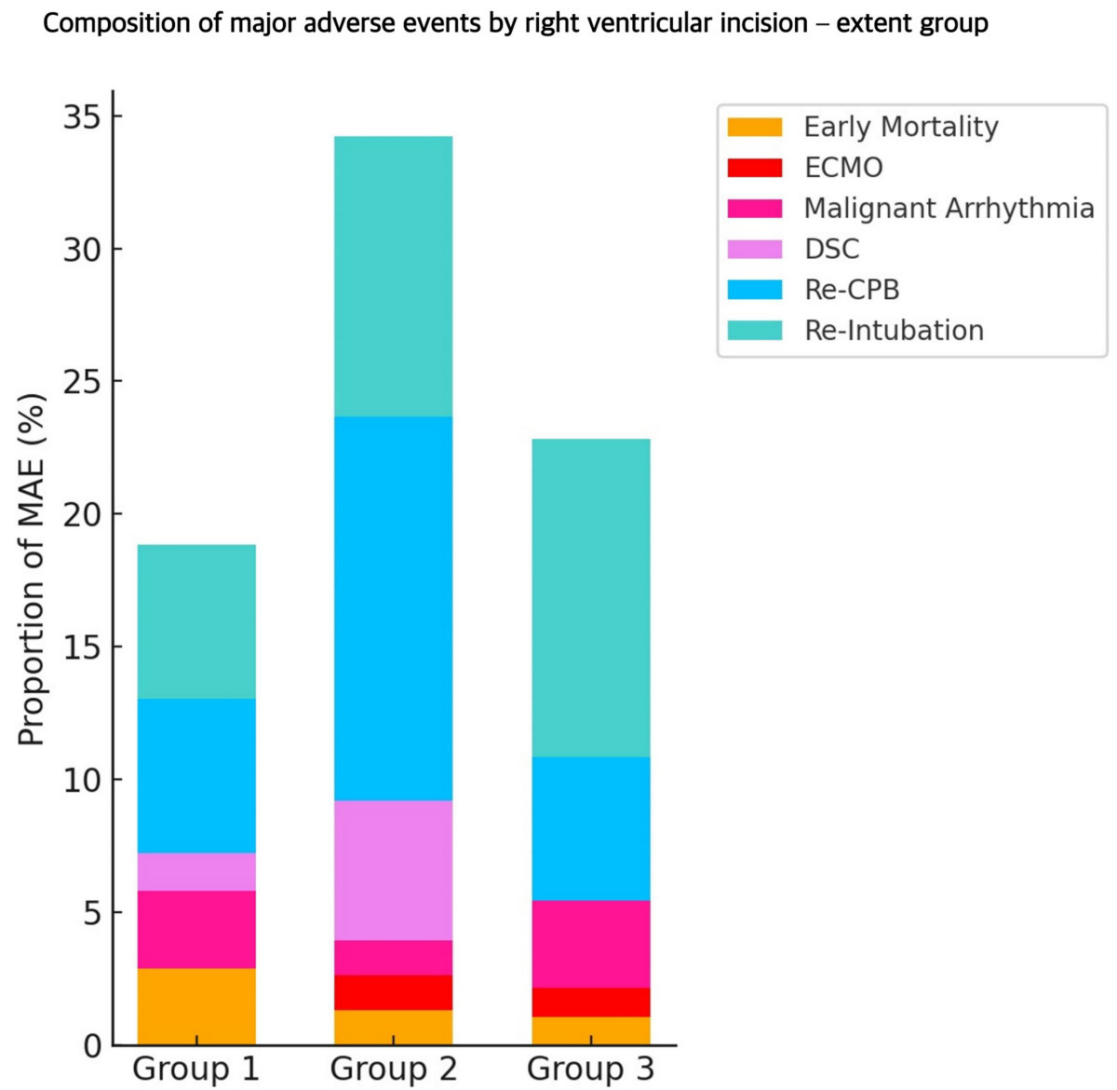
Stacked bar chart showing the distribution of individual adverse events contributing to the overall MAE rate in Group 1 (no RV incision), Group 2 (incision within the infundibulum), and Group 3 (incision beyond the infundibulum). MAE was defined as a composite of early mortality, extracorporeal membrane oxygenation (ECMO), malignant arrhythmias, delayed sternal closure (DSC), reoperation with cardiopulmonary bypass (Re-CPB), and reintubation. While the overall incidence of MAE was comparable across groups, the relative contribution of specific complications differed, with higher proportions of DSC and Re-CPB observed in Group 2 and more frequent reintubation in Group 3.

Major adverse events (MAEs) occurred in 44 patients (18.6%): 9 in Group 1, 19 in Group 2, and 16 in Group 3 (*P* = 0.184, Table 3). No significant differences were observed among groups with respect to mortality, extracorporeal membrane oxygenation, malignant arrhythmias, delayed sternal closure, reoperation, or reintubation, both before and after propensity score matching. MAE rates are shown in Figure 2, and the composition of MAEs is summarized in Figure 3.

**Table 3:** Postoperative date.

Variables	Overall	No incision	Within infundibulum	Beyond infundibulum	*p* value
MAO (%)	44 (18.6)	9 (13.0)	19 (25.0)	16 (17.4)	0.184
Early mortality (%)	4 (1.7)	2 (2.9)	1 (1.3)	1 (1.1)	0.688
ECMO (%)	2 (0.8)	0 (0.0)	1 (1.3)	1 (1.1)	1
Malignant arrhythmia (%)	6 (2.5)	2 (2.9)	1 (1.3)	3 (3.3)	0.772
DSC (%)	5 (2.1)	1 (1.4)	4 (5.3)	0 (0.0)	0.052
Re CPB (%)	20 (8.4)	4 (5.8)	11 (14.5)	5 (5.4)	0.09
Reintubation (%)	23 (9.7)	4 (5.8)	8 (10.5)	11 (12.0)	0.403
Elevated CVP (%)	56 (23.6)	11 (15.9)	23 (30.3)	22 (23.9)	0.122
ICU stay [median (IQR)]	89.2 [46.5, 165.6]	96.0 [48.0, 144.0]	120.0 [46.8, 198.0]	69.0 [42.6, 138.9]	0.011
Hospital stay [median (IQR)]	13.0 [9.0, 20.0]	8.0 [7.0, 11.0]	13.0 [9.0, 16.2]	19.0 [13.0, 25.0]	<0.001
Intubation time [median (IQR)]	17.2 [6.8, 67.0]	10.0 [4.0, 25.0]	24.8 [7.0, 102.5]	21.4 [8.4, 52.3]	0.001
Postop 24 h Drainage [median (IQR)]	140.0 [100.0, 219.0]	160.0 [116.0, 315.0]	146.5 [90.0, 230.0]	125.0 [95.0, 170.5]	0.011
Postop 48 h blood product (%)	118 (49.8)	18 (26.1)	36 (47.4)	64 (69.6)	<0.001
Chylothorax (%)	8 (3.4)	1 (1.4)	2 (2.6)	5 (5.4)	0.396
Infections (%)	20 (8.4)	2 (2.9)	3 (3.9)	15 (16.3)	0.004
Diaphragmatic paralysis (%)	4 (1.7)	0 (0.0)	1 (1.3)	3 (3.3)	0.39

### Secondary outcomes

Secondary outcomes are summarized in Table 3 and Supplementary Table S3. In crude analyses, ventilation time, length of ICU stay, and total hospital stay differed significantly among groups (all *P*'s < 0.05, Kruskal–Wallis test). Dunn's *post-hoc* tests with Bonferroni correction confirmed that Group 1 had shorter durations than both Groups 2 and 3, whereas no significant differences were observed between Groups 2 and 3. Although not reaching statistical significance, delayed sternal closure was more frequently observed in Group 2 than in Group 1 (5.3% vs. 1.4%, *P* = 0.052), suggesting a potential trend toward increased perioperative complexity associated with infundibular incisions. In contrast, Group 3 demonstrated significantly higher transfusion requirements and postoperative infection rates compared with both Groups 1 and 2 (*P* < 0.01 for both), indicating a greater inflammatory and hemodynamic burden in patients with more extensive right ventricular incisions. After propensity score matching, delayed sternal closure and postoperative 24-h drainage remained significantly different between Groups 2 and 3 (*P* = 0.046 and *P* = 0.038, respectively). In contrast, transfusion requirements were no longer statistically significant (*P* = 0.137). Differences in length of ICU stay, ventilation duration, total hospital stay, and residual RVOT gradients were also no longer observed following adjustment.

## Discussion

The optimal extent of RV incision during repair of TOF remains a subject of debate. Although RV incision facilitates ventricular septal defect closure and relief of RVOT obstruction, concerns persist regarding postoperative ventricular dilation, dysfunction, and arrhythmias. Large registry data, including those from the Society of Thoracic Surgeons (STS) and the European Association for Cardio-Thoracic Surgery (EACTS) databases, indicate that RV incision remains the predominant approach worldwide; however, outcomes associated with different strategies remain inconsistent.

In our study, patients who underwent repair without an RV incision (Group 1) demonstrated more favorable early outcomes. These differences, however, were largely attributable to baseline anatomic advantages—older age, larger body size, and more favorable pulmonary valve and artery development—rather than an intrinsic superiority of the approach itself. Similar selection bias has also been noted in prior reports. Nevertheless, recent multicenter evidence supports the potential benefits of minimizing RV incision when feasible. Avşar et al. (28) reported that transatrial repair with pulmonary valve formation achieved better early outcomes compared with conventional RV incision repair, reinforcing the concept that avoiding or limiting RV incision may reduce perioperative morbidity in appropriately selected patients.

Groups 2 and 3, in which RV incisions were employed, exhibited distinct risk patterns. Patients with incisions confined to the infundibulum (Group 2) experienced longer operative times and a trend toward increased delayed sternal closure (5.3% vs. 1.4%, *P* = 0.052), whereas those with incisions extending beyond the infundibulum (Group 3) demonstrated higher transfusion requirements and postoperative infection rates. Despite these differences in secondary outcomes, the incidence of major adverse events did not differ significantly among groups after adjustment for baseline characteristics. These findings suggest that while RV incision is associated with increased perioperative complexity and morbidity, it does not necessarily translate into higher early mortality or catastrophic complications.

Our findings align with previous single-center retrospective studies that have emphasized the tradeoff between surgical exposure and long-term RV integrity. The novelty of our work lies in its multicenter design and the use of propensity score matching to reduce baseline imbalances, thereby providing a more reliable estimate of the short-term consequences of different incision strategies. Importantly, these results underscore that surgical decision-making should prioritize complete RVOT relief to ensure procedural safety while minimizing RV incision whenever technically feasible.

Tetralogy of Fallot represents a broad anatomical spectrum, with variability in pulmonary valve annulus size, infundibular morphology, and timing of clinical manifestation (23, 28). These factors critically influence the choice of surgical technique, including the need for palliative procedures prior to complete repair and the decision to use a transannular patch or perform a right ventricular incision (29). The primary surgical goal remains complete and effective relief of RVOT obstruction, as residual outflow tract obstruction has been shown to contribute to RV hypertrophy, dysfunction, and long-term reintervention (30). While efforts to preserve the pulmonary valve and avoid right ventriculotomy are desirable, this must not come at the expense of inadequate RVOT relief (24). When a transannular patch or an extensive incision is required to achieve optimal outflow, it should be performed decisively, with adequate counseling regarding potential risks.

Prior studies have shown that right ventricular incision disrupts myocardial architecture and conduction pathways, potentially increasing the long-term risk of arrhythmias and right ventricular dysfunction (14). In contrast, valve-sparing and transatrial–transpulmonary approaches may confer superior long-term preservation of RV function (31), although these techniques are feasible only in patients with favorable anatomy. Thus, surgical strategy should be tailored to each patient, considering anatomical severity (32), valve morphology, and pulmonary artery development. While the degree of RV incision should be minimized when feasible, this should not come at the expense of residual outflow tract obstruction or increased surgical risk. This individualized approach reflects current best practices and is supported by long-term follow-up data in adult survivors of TOF repair.

### Clinical implications

Our results provide several practical insights into the surgical management of TOF. First, the observation that patients who underwent repair without an RV incision experienced more favorable early outcomes emphasizes the importance of patient selection, as individuals with more favorable pulmonary valve and artery anatomy are most likely to benefit from a transatrial–transpulmonary approach. Second, the distinct perioperative risk profiles associated with limited vs. extended RV incisions suggest that the extent of incision should be individualized according to intraoperative anatomy, balancing the need for adequate exposure against the risk of increased morbidity. Finally, the absence of significant differences in major adverse events across groups reinforces that complete RVOT relief must remain the overriding surgical priority, with incision minimization serving as a secondary but meaningful goal. Together, these considerations may assist surgeons in refining operative strategies and in counseling families regarding the relative risks and benefits of different approaches.

### Limitations

This study has several limitations. First, its retrospective and observational design is inherently subject to selection bias and residual confounding, even after the use of propensity score matching. Second, although the analysis included two high-volume tertiary centers, center-specific practices and surgeon preferences may have influenced operative strategies and outcomes. Third, the study focused only on early postoperative outcomes; long-term follow-up data, including echocardiographic or cardiac magnetic resonance evaluation of RV function, pulmonary regurgitation, and late arrhythmias, were not available. As a result, we could not determine the impact of different RV incision strategies on long-term ventricular remodeling, valve competence, or reintervention risk. Finally, although relatively large for a congenital population, the study cohort remains modest compared with national registries, underscoring the need for prospective multicenter studies to validate these findings.

## Conclusions

In this multicenter retrospective study, the extent of RV incision during TOF repair was associated with distinct perioperative risk profiles, although rates of major adverse events did not differ significantly after adjustment for baseline imbalances. The more favorable outcomes observed in patients without an RV incision primarily reflected underlying anatomic advantages rather than the superiority of one approach over the other. These findings suggest that RV incision should be minimized whenever technically feasible; however, adequate RVOT relief must remain the priority to ensure procedural safety. Prospective, multicenter studies with long-term imaging-based follow-up are warranted to clarify the impact of incision strategy on ventricular remodeling, pulmonary regurgitation, and late clinical outcomes.

## Data Availability

The original contributions presented in the study are included in the article/Supplementary Material, further inquiries can be directed to the corresponding authors.
