# Supplementary material for: Impact of right ventricular incision extent on early outcomes after tetralogy of Fallot repair: a two-center retrospective cohort study
Source: Front Cardiovasc Med. 2026 Jan 23;12:1702538. doi: 10.3389/fcvm.2025.1702538 (PMC12876232; doi:10.3389/fcvm.2025.1702538)
Supplement: Supplementary file 1 [file Datasheet1.docx]

**Table 1S:Baseline characteristics after propensity score matching**

| **Variables** | **Overall** | **No Incision** | **Within Infundibulum** | **Beyond Infundibulum** | **p value** |
| --- | --- | --- | --- | --- | --- |
| n | 50.9 | 14.5 | 19.9 | 16.6 |  |
| **Baseline Characteristics** | NA | NA | NA | NA | NA |
| Male (%) | 33.8 (66.4) | 10.3 (71.1) | 13.7 (68.9) | 9.8 (59.4) | 0.623 |
| Age (median [IQR]) | 9.8 [7.0, 18.4] | 10.8 [8.0, 15.6] | 9.5 [6.3, 20.1] | 9.0 [6.0, 15.3] | 0.417 |
| Height (median [IQR]) | 70.0 [68.0, 80.0] | 69.7 [68.0, 76.0] | 70.0 [66.0, 80.0] | 70.0 [67.9, 80.6] | 0.961 |
| Weight (median [IQR]) | 8.0 [7.5, 9.5] | 8.0 [7.5, 9.8] | 8.0 [7.2, 9.4] | 8.0 [6.6, 9.0] | 0.648 |
| BSA (median [IQR]) | 0.4 [0.4, 0.5] | 0.4 [0.4, 0.5] | 0.4 [0.4, 0.5] | 0.4 [0.4, 0.4] | 0.87 |
| SPO2 (median [IQR]) | 93.0 [86.0, 97.0] | 90.8 [86.4, 96.0] | 93.0 [84.0, 96.0] | 95.0 [85.0, 98.0] | 0.556 |
| Coronary_Abnormalities (%) | 0.0 (0.0) | 0.0 (0.0) | 0.0 (0.0) | 0.0 (0.0) | 0.475 |
| Z_score (median [IQR]) | -2.4 [-3.7, -1.3] | -2.6 [-4.1, -1.5] | -1.8 [-2.9, -1.2] | -2.8 [-3.7, -1.1] | 0.417 |
| Nakata (median [IQR]) | 226.8 [157.9, 285.9] | 189.5 [155.1, 253.2] | 231.1 [172.0, 315.8] | 224.1 [144.4, 284.6] | 0.768 |
| Mcgoon (median [IQR]) | 2.2 [1.7, 2.7] | 1.9 [1.7, 2.4] | 2.3 [1.8, 2.6] | 2.5 [1.8, 2.7] | 0.461 |

**Table 2S:Operative and perioperative outcomes after propensity score matching**

| **Variables** | **Overall** | **No Incision** | **Within Infundibulum** | **Beyond Infundibulum** | **p value** |
| --- | --- | --- | --- | --- | --- |
| TAP (%) | 20.1 (39.5) | 0.0 (0.0) | 10.9 (54.9) | 9.2 (55.6) | <0.001 |
| Extended_Incision (%) |  |  |  |  | NA |
| No Cut | 14.5 (28.4) | 14.5 (100.0) | 0.0 (0.0) | 0.0 (0.0) |  |
| Within infundibulum | 19.9 (39.0) | 0.0 (0.0) | 19.9 (100.0) | 0.0 (0.0) |  |
| Beyond infundibulum | 16.6 (32.6) | 0.0 (0.0) | 0.0 (0.0) | 16.6 (100.0) |  |
| VSD Repair Approach (%) |  |  |  |  | 0.001 |
| RA | 33.4 (65.6) | 14.1 (97.7) | 11.3 (56.7) | 8.0 (48.3) |  |
| RV | 11.7 (23.0) | 0.0 (0.0) | 4.5 (22.9) | 7.1 (43.1) |  |
| RA+RV | 5.5 (10.8) | 0.0 (0.0) | 4.1 (20.4) | 1.4 (8.6) |  |
| PA | 0.3 (0.7) | 0.3 (2.3) | 0.0 (0.0) | 0.0 (0.0) |  |
| CPB Time (median [IQR]) | 109.0 [94.0, 132.0] | 103.3 [94.0, 110.9] | 115.0 [102.1, 132.0] | 116.3 [88.2, 139.4] | 0.171 |
| ACC Time (median [IQR]) | 76.0 [58.6, 91.0] | 65.0 [58.3, 87.0] | 81.0 [74.0, 91.7] | 64.6 [50.9, 95.0] | 0.071 |
| RV Pressure (median [IQR]) | 40.0 [32.0, 47.0] | 37.9 [30.9, 42.0] | 40.0 [33.0, 45.5] | 40.0 [29.9, 50.0] | 0.656 |
| MPA Pressure (median [IQR]) | 24.0 [18.0, 29.0] | 22.8 [16.3, 25.1] | 21.7 [18.0, 29.9] | 26.0 [19.0, 31.2] | 0.152 |
| System Pressure (mean (SD)) | 78.8 (15.2) | 78.5 (14.0) | 78.0 (14.1) | 79.9 (17.5) | 0.908 |

**Table 3s:Postoperative Date after propensity score matching**

| **Variables** | **Overall** | **No Incision** | **Within Infundibulum** | **Beyond Infundibulum** | **p value** |
| --- | --- | --- | --- | --- | --- |
| MAO (%) | 11.9 (23.4) | 4.1 (28.4) | 4.6 (23.2) | 3.2 (19.4) | 0.769 |
| Early Mortality (%) | 1.4 (2.8) | 0.1 (0.6) | 0.8 (4.0) | 0.5 (3.3) | 0.549 |
| ECMO (%) | 1.2 (2.3) | 0.0 (0.0) | 0.8 (4.0) | 0.4 (2.5) | 0.599 |
| Malignant Arrhythmia (%) | 0.3 (0.6) | 0.3 (1.9) | 0.0 (0.0) | 0.0 (0.1) | <0.001 |
| DSC (%) | 0.7 (1.3) | 0.0 (0.0) | 0.7 (3.4) | 0.0 (0.0) | 0.046 |
| Re CPB (%) | 7.0 (13.7) | 1.3 (8.8) | 2.4 (12.1) | 3.3 (19.8) | 0.529 |
| Re Intubation (%) | 5.1 (10.0) | 2.6 (17.7) | 2.2 (10.9) | 0.3 (2.1) | 0.099 |
| Elevated CVP (%) | 9.7 (19.1) | 2.7 (18.6) | 4.0 (20.3) | 3.0 (18.1) | 0.969 |
| ICU Stay (median [IQR]) | 88.3 [44.1, 165.0] | 118.3 [72.0, 219.7] | 48.0 [41.3, 123.7] | 69.4 [35.9, 142.4] | 0.009 |
| Hospital Stay (median [IQR]) | 13.0 [11.0, 18.0] | 11.0 [8.0, 15.0] | 13.0 [11.4, 17.4] | 14.2 [11.0, 19.0] | 0.11 |
| Intubation Time (median [IQR]) | 20.0 [6.4, 76.3] | 21.1 [5.0, 91.9] | 23.5 [6.0, 75.0] | 12.8 [6.9, 37.4] | 0.903 |
| Postop 24H Drainage (median [IQR]) | 125.0 [88.0, 188.7] | 157.6 [112.3, 214.4] | 94.8 [75.0, 170.0] | 125.0 [95.0, 182.7] | 0.038 |
| Postop 48H Blood Product (%) | 22.5 (44.3) | 4.2 (29.2) | 8.7 (43.9) | 9.6 (57.8) | 0.137 |
| Chylothorax (%) | 1.2 (2.4) | 0.0 (0.3) | 0.5 (2.5) | 0.7 (4.2) | 0.282 |
| Infections (%) | 2.4 (4.7) | 0.4 (2.6) | 1.4 (7.1) | 0.6 (3.6) | 0.504 |
| Diaphragmatic_Paralysis (%) | 0.3 (0.5) | 0.0 (0.0) | 0.2 (1.1) | 0.1 (0.4) | 0.488 |
